# Pituitary Metastasis in Lung Cancer Patients: Case Series and Review of the Literature

**DOI:** 10.3390/curroncol33060362

**Published:** 2026-06-16

**Authors:** Sofia Ntouraki, Afroditi Roumpou, Athina Asimakopoulou, Ioannis Gkiozos, Fotini Sarropoulou, Maria Mani, Androniki Marioli, Dimitrios Bouklas, Konstantinos Syrigos, Melpomeni Peppa

**Affiliations:** 1 Oncology Unit, Third Department of Internal Medicine, Sotiria General Hospital for Chest Diseases, School of Medicine, National and Kapodistrian University of Athens, 11527 Athens, Greeceathasimak@med.uoa.gr (A.A.); yiannisgk@hotmail.com (I.G.); sphotini@hotmail.com (F.S.); marilimanimd@gmail.com (M.M.); ksyrigos@med.uoa.gr (K.S.); 2 Endocrine Unit, Second Propaedeutic Department of Internal Medicine, Attikon University Hospital, School of Medicine, National and Kapodistrian University of Athens, 12462 Athens, Greece; afroditiroum@gmail.com; 3 Oncology Unit, Athens Medical Group, 15125 Athens, Greece; nmarioli@yahoo.gr; 4 Department of Pathology, Georgios Gennimatas General Hospital, 11527 Athens, Greece; dpbouklas@outlook.com.gr

**Keywords:** lung cancer, pituitary mass, immunotherapy, hypopituitarism, case series

## Abstract

The pituitary gland is an uncommon site of tumor metastasis and is predominantly associated with malignancies of the lung and breast. Metastatic involvement of the pituitary gland in lung cancer (LC) typically indicates advanced disease and is associated with poor prognosis and pituitary insufficiency, which often remains underdiagnosed and significantly affects quality of life and survival. We present four cases of pituitary metastasis (PM) originating from LC, characterized by distinct histological subtypes, variable timing from initial diagnosis, and diverse clinical manifestations. Clinical presentation was heterogeneous: two patients had involvement of both pituitary lobes with multiple pituitary hormone deficiencies, one had anterior lobe involvement with anterior pituitary deficiency following immune checkpoint inhibitor-associated hypophysitis, and one remained asymptomatic. Therapeutic approaches included partial surgical resection followed by radiotherapy in two patients and radiotherapy alone in the other two; all patients continued systemic antineoplastic therapy and received hormone replacement as indicated. Mean overall survival was 7.5 months. PM can occur across all histological subtypes of LC and typically signifies advanced disease with poor prognosis. Early identification and appropriate management of hypopituitarism may improve quality of life and clinical outcomes.

## Simple Summary

The pituitary gland is an uncommon site of tumor metastasis; however, lung and breast cancers have most frequently been reported as the primary malignancy. In lung cancer, pituitary metastasis usually occurs in advanced stages of the disease, typically linked to poor prognosis. It is often underdiagnosed due to its absent or non-specific symptomatology, although it may lead to significant hormone deficiencies. Herein, we present four cases of pituitary metastasis from lung cancer, having different histological subtypes, timing of presentation, and clinical manifestations. The presentation varied from multiple pituitary hormone deficiencies to few or no symptoms. Treatment included surgery, radiotherapy, systemic anticancer therapy, and hormone replacement when needed. The description of these cases highlights the importance of early recognition and appropriate management of pituitary metastasis, aiming to improve patients’ overall functional status and clinical outcomes.

## 1. Introduction

The pituitary gland is an uncommon site of metastasis, corresponding to <1% of all intracranial secondary tumors [1]. Pituitary metastasis (PM) is often subclinical and/or underestimated due to the rarity and the lack of clinical awareness [2]. However, its incidence seems to be increased due to population aging, broader use of imaging modalities, better knowledge of the endocrine side effects of cancer and its treatments, and improved survival of patients undergoing advanced therapeutic interventions for their primary malignancy [3].

PMs have been documented across different types of cancer. Lung cancer (LC) is the most prevalent primary cancer, accounting for up to 46% of all cases in males, while breast cancer ranks first in females, as it is the cause of up to 50% of PMs. Combined breast cancer and LC account for 60% of all PM reports [1], which mostly present at a mean age of approximately 60 years [1].

The majority of PMs are clinically silent. Most cases are incidentally discovered in neuroimaging or during autopsy [4]. Symptoms are reported in <20% of cases and they are usually non-specific (fatigue, loss of appetite, headache, nausea) [1].

PM diagnosis is challenging as its symptoms are often masked by the clinical signs of LC and its treatments [1]. Furthermore, differentiating PM from other pituitary tumors or from immune checkpoint inhibitor (ICI)-induced hypophysitis is challenging, as they share common clinical presentations and overlapping MRI features [5,6]. However, manifestations related to local mass effect have been reported, such as visual field defects, headaches, and compression of adjacent structures (e.g., cranial nerve palsies), alongside symptoms of posterior and—less commonly—anterior pituitary insufficiency [7,8,9].

Therapeutic management of the PM may include surgical resection, which is mostly non-curative; radiotherapy; and systemic treatments [2,10]. The prognosis of patients with PM is generally unfavorable and is even worse in cases of metastatic LC [1,11]. PMs typically occur in the context of advanced disease [11], and mortality is primarily driven by systemic metastatic progression rather than the pituitary lesion itself [1].

The aim of this paper is to present four cases of PM arising from different histological subtypes of LC, with different clinical presentation, highlighting the challenges in diagnosis and treatment, and their impact on overall survival. A review of the growing body of literature on this important topic has also been conducted.

## 2. Case Presentation

All patients were clinically managed and followed up at the Oncology Unit of the Third Department of Internal Medicine in ‘Sotiria’ General Hospital for Chest Diseases in Athens, Greece. Treatment decisions were guided by a multidisciplinary team of oncologists, endocrinologists, and pathologists. Clinical, radiological, and laboratory data were retrospectively collected from institutional health records. Throughout this study, patient anonymity and the confidentiality of personal data were strictly maintained.

Patients’ personal and disease characteristics are summarized in Table 1.

Laboratory exams of the patients at the time of pituitary metastasis diagnosis are summarized in Table 2.

### 2.1. Case 1

In September 2018, a 64-year-old female patient was referred to our oncology department due to a neuroendocrine tumor, confirmed with fine needle aspiration of a palpable, protruding, submandibular lymph node. The patient was a painter, ex-smoker of 40 pack-years, and had a medical history of hypertension and type 2 diabetes mellitus under treatment. A diagnostic staging computed tomography (CT) scan revealed foci in both lungs, the liver, and mediastinal and submandibular lymph nodes. The subsequent bronchoscopic lung biopsy and hepatic FNA confirmed the diagnosis of a neuroendocrine neoplasm of lung origin, namely small cell LC (SCLC), stage IVB. She received first-line chemotherapy with carboplatin and etoposide for five months. Due to disease progression, treatment was subsequently switched to ICI therapy with nivolumab, which was continued for 18 months. Because of aggravated weakness, severe hypotension and visual field defects, the patient was admitted to the hospital. Laboratory evaluation revealed multiple anterior pituitary insufficiency (TSH, ACTH, FSH, and LH deficiencies) and hyperprolactinemia (Table 2). Visual field testing confirmed bitemporal hemianopsia. Brain MRI identified a pituitary mass measuring up to 1.5 cm in diameter, extending into the suprasellar region, exerting pressure on the optic chiasm. During hospitalization she received glucocorticoids intravenously, and she was discharged with per os hydrocortisone and levothyroxine replacement therapy. A few weeks later the patient complained of severe polyuria and polydipsia. New laboratory evaluation revealed plasma hyperosmolality and urine hypoosmolality (Table 2), consistent with arginine vasopressin deficiency (AVP-D; previously known as central diabetes insipidus), and desmopressin replacement therapy was initiated. Repeat brain MRI showed a slight increase in pituitary mass dimensions. The patient underwent partial transsphenoidal excision of the mass and histopathological examination revealed a diffusely infiltrated, metastatic, malignant neuroendocrine carcinoma of lung origin (immunohistochemistry positive for cytokeratin (CK) 8–18, synaptophysin, chromogranin, and Thyroid Transcription Factor-1 positivity [12]) (Figure 1).

In parallel, she underwent adjuvant radiation therapy targeted at the sellar region. Visual fields gradually improved, and the dimensions of the PM were reduced. Due to disease progression, she was initiated on third-line chemotherapy with irinotecan. The patient’s overall survival was 43 months from her initial cancer diagnosis and 6 months from the diagnosis of PM. Complete history of case 1 is summarized as a timeline in Figure 2.

### 2.2. Case 2

In October 2022, a 63-year-old male patient, ex-smoker of 35 pack-years, with no medical history, was referred for further evaluation of an incidentally detected left lower-lobe mass on chest radiograph. Chest CT revealed multiple masses in both the left lower and right upper pulmonary lobes, as well as a few suspicious mediastinal lymph nodes. Positron Emission Tomography/Computed Tomography (PET/CT) demonstrated some hypermetabolic foci in the lungs and left hip bone, along with some hypermetabolic mediastinal and pelvic lymph nodes. Brain CT showed no evidence of neoplastic disease. The bronchoscopic lung biopsy revealed adenocarcinoma, namely non-small cell lung cancer (NSCLC), stage IVB. Comprehensive molecular analysis using next-generation sequencing did not identify actionable mutations, and PD-L1 expression was found to be <1%. The patient was initiated on first-line treatment with chemo-immunotherapy (carboplatin-pemetrexed-nivolumab-ipilimumab), followed by ICI treatment (nivolumab-ipilimumab). After receiving 5 cycles of nivolumab-ipilimumab, he complained of fatigue, dizziness, and severe headaches. Clinical examination revealed hypotension (Blood Pressure: 80/55 mmHg). Ophthalmological examination was normal. Laboratory tests indicated multiple anterior pituitary insufficiency (TSH, ACTH, FSH and LH deficiencies). Brain MRI depicted slight homogeneous pituitary and stalk enlargement, with diffuse enhancement, without bone destruction. Based on the clinical presentation, hormonal profile, and brain imaging findings, a diagnosis of grade 3 immune-induced hypophysitis secondary to immunotherapy was established. Immunotherapy was withheld, and the patient was admitted to the hospital. After the intravenous administration of hydrocortisone, the patient’s symptoms gradually alleviated, and a week later he was discharged with per os hydrocortisone and levothyroxine replacement therapy. Antineoplastic treatment was resumed a month later, with the discontinuation of ipilimumab, while nivolumab was continued as maintenance therapy, as hypophysitis is primarily attributed to the administration of CTLA-4 inhibitors (ipilimumab) [6]. The patient was asymptomatic and retained a good performance status (score of 0–1 according to the Eastern Cooperative Oncology Group, ECOG) for the next four months. However, at the end of this period, follow-up chest CT revealed progression of the disease, so he was started on second-line treatment with docetaxel and nintedanib. Two months later, the patient exhibited a relapse of symptoms involving severe cephalalgia, light headedness, weakness, and visual field defects. Hormonal evaluation at that time once again demonstrated the known multiple anterior pituitary insufficiency (Table 2). Pituitary MRI depicted a dumbbell-shaped lesion centered in the pituitary sella, measuring approximately 20 × 22 × 31 mm, with suprasellar extension and compression of the optic chiasm (Figure 3).

Visual field testing revealed bitemporal hemianopsia. The mass was partially excised via endoscopic transsphenoidal surgery. Histopathological assessment revealed a metastatic adenocarcinoma of moderate differentiation, exhibiting immunohistochemical markers indicative of pulmonary origin (napsin, TTF-1 positivity [13]), as well as an elevated level of inflammatory infiltration, probably indicating the former diagnosis of hypophysitis (Figure 4).

Palliative treatment with local radiotherapy and chemotherapy was initiated. The patient’s overall survival was 18 months from the initial cancer diagnosis and 6 months from the diagnosis of PM. Complete history of case 2 is summarized as a timeline in Figure 5.

### 2.3. Case 3

In December 2015, a 64-year-old male patient, current smoker with a 60–pack-year history and treated hypertension and dyslipidemia, was diagnosed with a lung mass after chest CT performed for hemoptysis, and referred for oncologic assessment. The subsequent PET/CT imaging revealed hypermetabolic activity in the left upper lung lobe, the right and left lung hila, the mediastinal, subclavian, and supraclavicular lymph nodes, as well as in the lumbar vertebrae. Brain MRI revealed no evidence of neoplastic disease. The bronchoscopic lung biopsy set the diagnosis of squamous NSCLC, stage IVB. Molecular testing revealed a negative next-generation sequencing profile and <1% of PD-L1 expression. Chemotherapy with cisplatin and vinorelbine was initiated, followed by second-line immunotherapy with nivolumab, due to a new metastatic lesion in the right kidney. Ten months later, although the patient was asymptomatic, laboratory tests were indicative of thyrotoxicosis [TSH 0.02 μIU/mL, FT4 2.11 ng/dL (normal values: 0.35–4.45 μIU/mL, 0.93–1.7 ng/dL, respectively)], which was later followed by hypothyroidism [TSH 11.87 μIU/mL, FT4 0.56 ng/dL (normal values: 0.35–4.45 μIU/mL, 0.93–1.7 ng/dL, respectively)]; the rest of the pituitary hormones were normal. ICI-treatment-induced thyroiditis was suspected, and levothyroxine therapy was initiated, leading to gradual normalization of thyroid function [TSH 3.29 μIU/mL, T4 1.06 ng/dL (normal values: 0.35–4.45 μIU/mL, 0.8–1.9 ng/dL, respectively)]. After seven months, the patient was asymptomatic, his anterior pituitary function was normal (Table 2) and he underwent restaging with brain MRI, which depicted a PM in the left side of the pituitary gland, extending to the left cavernous sinus (maximum diameter, 1.3 cm), without affecting the optic chiasm. Due to progression of the disease, ICI treatment was interrupted, and the patient underwent Cyber Knife radiosurgery. The post-radiation brain MRI showed reduction in the mass. Third-line treatment with docetaxel was administered for three months; however, the lung disease burden continued to increase, prompting a switch to fourth-line treatment with carboplatin and gemcitabine, which was given for an additional five months. The patient’s overall survival was 26 months from the initial cancer diagnosis and 12 months from the diagnosis of PM. Complete history of case 3 is summarized as a timeline in Figure 6.

### 2.4. Case 4

In February 2022, a 65-year-old female patient, with a history of papillary thyroid carcinoma at the age of 45 years, was referred for evaluation of an incidentally identified pulmonary mass on chest CT. The subsequent PET/CT imaging revealed hypermetabolic activity in the left lower and upper lung lobes, right lung hilum, and mediastinal and supraclavicular lymph nodes. Brain MRI revealed a benign meningioma in the right ventricle, with no additional lesions. The transbronchial needle biopsy of the right lower paratracheal and subcarinal lymph nodes revealed adenocarcinoma (NSCLC), while molecular testing identified a mutation in exon 21 of the epidermal growth factor receptor (EGFR). The patient was diagnosed with NSCLC stage IV and was initiated on targeted anticancer therapy with osimertinib. At baseline, she had an excellent performance status (ECOG 0). Her laboratory results were normal, and she was euthyroid under levothyroxine replacement treatment [TSH 3.28 μIU/mL, FΤ4 1.4 ng/dL (normal values: 0.35–4.94 μIU/mL and 0.7–1.48 ng/dL, respectively)]. After two years, brain MRI revealed multiple foci in the white matter of both cerebral hemispheres, indicating progression of the disease. Stereotactic radiosurgery was administered, and second-line treatment (carboplatin-pemetrexed-amivantamab) was initiated. Six months later, the patient presented with polyuria and polydipsia. The results of a 24 h urine analysis showed a urine volume of 9700 mL (>50 mL/kg/24 h), with low osmolality (102 mOsm/kg), consistent with AVP-D. Multiple pituitary insufficiency (TSH, ACTH, FSH and LH deficiencies) and hyperprolactinemia were also found (Table 2). Brain MRI depicted a new focus in the right occipital lobe, enlargement of the pituitary gland and stalk (measured 1.8 × 0.8 cm), and compression of the optic chiasm (Figure 7).

The patient was referred for brain radiotherapy. Hormonal replacement therapy with hydrocortisone and desmopressin was prescribed, and levothyroxine dose was titrated. The patient was enrolled in a clinical trial and received a third-line treatment with an antibody–drug conjugate. Despite achieving a partial response as per RECIST 1.1 imaging criteria (Response Evaluation Criteria in Solid Tumors) [14], her performance status progressively declined. She was subsequently hospitalized and passed four months following the initiation of third-line therapy. Her overall survival was 37 months from the initial diagnosis of cancer and 6 months from the diagnosis of PM. Complete history of case 4 is summarized as a timeline in Figure 8.

## 3. Discussion

Herein, we present four cases of patients with PM, arising from different histological types of LC, presenting at different intervals from the diagnosis of the primary tumor, and having various clinical presentations.

PM is rare but complicates the course of many cancer types, such as lung, breast, colon, prostate and renal cancer, melanoma, and hematological malignancies [15]. The most common primary cancers with PM are LC, which accounts for 23.7–46% in males, and breast cancer, responsible for 39.7–50% of cases in females [1,10,16,17].

Both types of LC, namely SCLC and NSCLC, have the potential to metastasize to the pituitary gland [18]. Although PM from neuroendocrine carcinomas is considered exceedingly rare [19], SCLC is recognized as the most common LC subtype associated with PM [5], owing to its aggressive nature and its significant metastatic potential, particularly to the central nervous system, as in our case 1 [8].

Our cases confirm that the clinical presentation of LC-associated PM can vary widely, depending on the site and the extent of the involvement, ranging from asymptomatic or non-specific symptoms (fatigue, vomiting, weight loss)—that are often misdiagnosed as they are similar to symptoms related to LC and its treatments—to mass effect symptoms (visual field defects, severe headaches, cranial nerve palsies) and severe pituitary insufficiency [10,20]. Atallah-Yunes et al. support that manifestations resulting from PM may serve as the initial clinical indication leading to the diagnosis of the malignancy, particularly in cases of SCLC, since LC typically gives symptoms no earlier than in the advanced stages of the disease [21].

AVP-D is the most prominent manifestation of LC-derived posterior pituitary lobe involvement, which is predominantly affected [22,23], as in our cases 1 and 4. The preferential involvement of the neurohypophysis can be explained by its direct vascularization via the hypophyseal arteries, and its extensive interface with the adjacent dura mater, as cancer cells can also spread to the pituitary gland from juxtasellar and base-of-skull metastases, or through meningeal spread by the suprasellar cistern [2,10]. However, AVP-D may remain clinically silent and manifest upon initiation of glucocorticoid therapy, as observed in patient 1 [8]. This is due to several mechanisms: cortisol induced relative resistance of the renal vasopressin 2 receptor, resulting in increased vasopressin release via upregulation of corticotropin-releasing hormone; renal sodium loss and volume depletion due to hypocortisolism; and decreased stroke volume and cardiac output in the context of glucocorticoid deficiency [24].

PM isolated in the anterior pituitary lobe is relatively uncommon, likely due to its primary supply via the portal venous system and secondary supply from the lower infundibular stem, which partially originates in the posterior lobe [2,10]. In a review of 201 cases of PM, McCormick et al. found that the posterior pituitary gland was involved in 84.6% of cases—either in isolation or along with the anterior lobe—while only 15.4% showed exclusive involvement of the anterior pituitary [25]. Secondary hypothyroidism and adrenal insufficiency represent the most prevalent manifestations of anterior pituitary involvement, followed by hypogonadism [23], as in our cases 1, 2 and 4. Metastatic infiltration of the anterior pituitary may also present with growth hormone and insulin-like growth factor 1 deficiencies, while, in rare cases of primary tumors with ectopic production of growth hormone-releasing hormone (GHRH), these can be found secondarily elevated [26]. In our cases 1, 2 and 4, growth hormone and insulin-like growth factor 1 levels remained within normal limits at the time of PM diagnosis. Hyperprolactinemia (cases 1 and 4) is also a potential manifestation of PM, with most cases resulting from the pituitary stalk effect [21].

Now that immunotherapy has been increasingly used in the treatment of LC, distinguishing the LC-associated PM from immune checkpoint inhibitor-induced hypophysitis poses a diagnostic challenge [6]. Both conditions can present with pituitary dysfunction, but, unlike PM, hypophysitis induced by ICI treatment primarily affects the anterior pituitary lobe [6]. Medical history, time of onset, imaging findings, and steroid response should be considered part of the differential diagnosis process [27]. Rarely, ICI-induced hypophysitis may also coincide with PM [28], as in our Case 2. In this patient, treatment with ICI was complicated by hypophysitis, which is an inflammatory state that induces structural changes in the pituitary gland, while glucocorticoid therapy further modified the local immune environment. Whether this intense, therapy-induced glandular inflammation increases the risk of subsequent PM remains a compelling theoretical question. Inflammatory microenvironments are known to increase local vascular permeability and upregulate endothelial adhesion molecules, theoretically creating a favorable “niche” that facilitates the homing and extravasation of circulating tumor cells into the pituitary stroma. Based on the studies supporting the role of the tumor microenvironment in tumor pathogenesis and aggressiveness [29,30,31,32], the above alterations may have increased the susceptibility of the anterior lobe to neoplastic infiltration. This could potentially explain the sequence of events, as well as the selective involvement of the anterior pituitary observed in Case 2.

The differentiation of PMs from primary pituitary tumors might be another challenge. Pituitary tumors represent approximately 15% of all intracranial neoplasms and pituitary incidentalomas, and they are detected more often than previously due to the increasing use of brain imaging modalities [5,25]. Most of the primary pituitary tumors are benign, while carcinomas are very rare; pituitary teratomas, germinomas, choriocarcinomas and gangliocytomas are even more rarely encountered [33,34]. Differentiating PMs from other pituitary tumors is based on the medical history and the neuroimaging. MRI is considered the most effective imaging modality, whereas a CT scan is not helpful [35]. Specific radiological features, including pituitary stalk thickening, cavernous sinus invasion, adjacent sella turcica sclerosis, or the presence of a distinctive “dumbbell-shaped” morphology of the lesion, may be indicative of metastatic involvement of the pituitary gland [22]. In addition, PMs seem to be smaller in size than adenomas, and most are isointense to brain parenchyma on T1- and T2-weighted images [35]. Rapid growth of a sellar tumor accompanied by aggressive invasion into surrounding tissues should also raise suspicion of PM [10]. However, the gold standard of diagnosis is the histopathological identification of the lesion [5,10]. In cases of well-differentiated neuroendocrine PMs, like those deriving from SCLC, differential diagnosis can be even more challenging, and a proper use of specific immunohistochemical stains may be needed [e.g., pituitary-specific transcription factors, such as pituitary-specific positive transcription factor 1 (Pit1), T-box transcription factor (T-PIT), steroidogenic factor 1 (SF-1), and GATA transcription factor 3 (GATA3), or TTF-1] [36].

PM management typically requires a multidisciplinary and individualized approach. Treatment decisions should be guided by the patient’s primary malignancy, the presence and severity of clinical symptoms, and the patient’s general status. The transsphenoidal approach aims to confirm the diagnosis, and/or decompress and alleviate symptoms related to mass effect (cases 1 and 2). However, it does not have a significant impact on survival [3,7]. Complete resection of the mass is usually impossible, due to the high diffusion, the invasion to the surrounding structures and the high vascularization of these tumors [1,2]. Radiotherapy, administered either as stereotactic radiation or whole-brain radiation, may be used either as a primary or adjuvant therapy [37]. Ιn recent years, targeted radiotherapy has correlated with a significant extension in survival time, as treated patients demonstrate a median survival of 16 months, compared to 6 months in those who remain untreated. Furthermore, stereotactic radiotherapy appears to yield superior outcomes when compared to conventional radiotherapy [1]. Systemic chemotherapy, ICI therapy, or targeted therapy is typically administered based on the specific characteristics of the primary tumor and the overall disease burden [7]. In cases of pituitary insufficiency, hormone replacement therapy should also be initiated [1], hierarchized as follows: first cortisol replacement, followed by thyroxine, and sex hormones. Prioritizing cortisol replacement is crucial to prevent unmasking a potentially life-threatening adrenal insufficiency and to avoid misinterpretation of hormone studies related to other untreated deficiencies [38].

The prognosis of PM is generally poor. The median survival time following diagnosis ranges between 6 and 22 months, with an overall median of 12.9 months [2]. This variation depends on factors such as the stage and histological type of the primary malignancy, the presence of additional metastases, the treatments administered, and the individual characteristics of the patient [2]. Prognosis seems to be worse when PM derives from LC in comparison to other primary tumors. According to a recent systematic review including 657 cases of PM, patients with LC exhibit shorter survival after PM diagnosis in comparison to patients with breast and kidney cancer (9 months in LC, 22 months in breast cancer, 30 months in kidney cancer) [11]. The poor survival rate observed in patients with LC is attributed to the aggressive nature of the disease and the high probability of extensive metastatic spread at the time of diagnosis [11]. In our cases, the median survival after the diagnosis of PMs was 7.5 months.

In this case series, we report four patients with PM originating from LC, encompassing all major histological subtypes and exhibiting a broad spectrum of clinical presentations. Three of the four patients exhibited pituitary insufficiency: two with combined anterior and posterior lobe involvement, and one with isolated anterior lobe deficiency (Case 2). Notably, the patient with isolated anterior lobe involvement had previously developed ICI-related hypophysitis several months before the detection of PM. This temporal sequence raises the hypothesis that prior inflammation may have rendered the anterior pituitary more susceptible to metastatic infiltration, although definitive clinical evidence is limited. Future studies with larger cohorts are warranted to validate these preliminary observations and to further elucidate the potential association between hypophysitis and increased vulnerability of the pituitary gland to metastatic seeding.

## 4. Conclusions

LC remains a significant global health challenge, despite improvements in prognosis following the advent of novel therapeutic modalities. PMs associated with LC, although rare, do occur and impose an additional burden on the primary disease. PMs can arise from all LC subtypes and typically indicate an advanced and aggressive disease. They most commonly cause posterior pituitary insufficiency but may also involve anterior pituitary dysfunction isolated or combined with posterior deficiency, and a range of specific or non-specific symptoms. The potential role of hypophysitis induced by ICI treatment to PM pathogenesis should be underlined. Diagnosis and differentiation from other pituitary tumors primarily rely on neuroimaging. Treatment mainly focuses on symptom control rather than cure. Healthcare professionals should be aware of LC-related PM in order to facilitate early recognition, tailored treatment, and optimization of the disease outcomes.

## Figures and Tables

**Figure 1:**
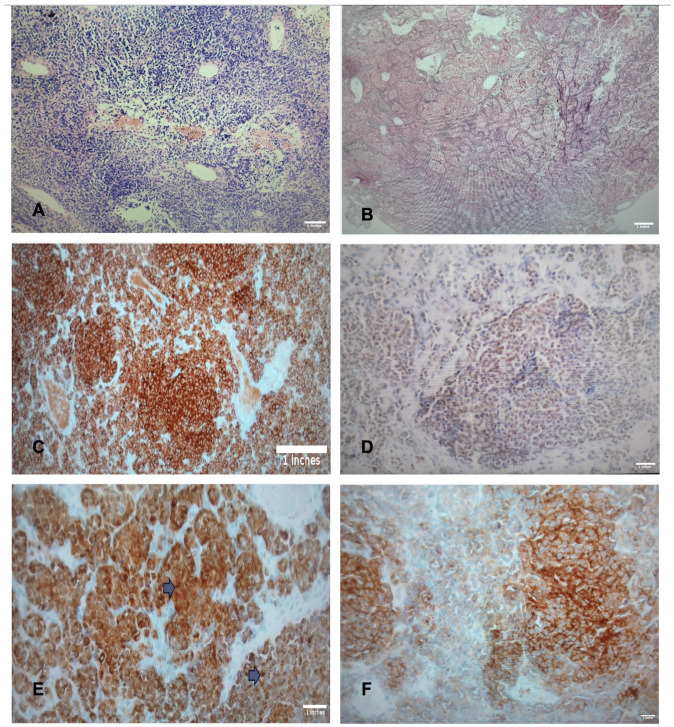
Histopathological and immunohistochemical features of the pituitary tumor in Case 1. Τhe white scale in the bottom right of each picture represents a scale bar of 1 inch. (**A**) Hematoxylin and eosin (H&E) stain (original magnification ×100) showing diffuse growth of small monomorphic cells with scant cytoplasm and round nuclei. (**B**) Reticulin stain (×40) demonstrating mostly preserved acinar architecture with an intact reticulin network. (**C**) Synaptophysin immunostaining (×40) with strong diffuse cytoplasmic positivity, supporting neuroendocrine differentiation. (**D**) Thyroid Transcription Factor-1 (TTF-1) immunostain (×200) showing weak to focally strong nuclear positivity. (**E**) Cytokeratin 8/18 (CK8/18) immunostain (×200) highlighting strong cytoplasmic positivity, with rare dot-like peri-nuclear accentuation (arrows). (**F**) Chromogranin A immunostain (×200) demonstrating patchy but strong cytoplasmic staining.

**Figure 2:**
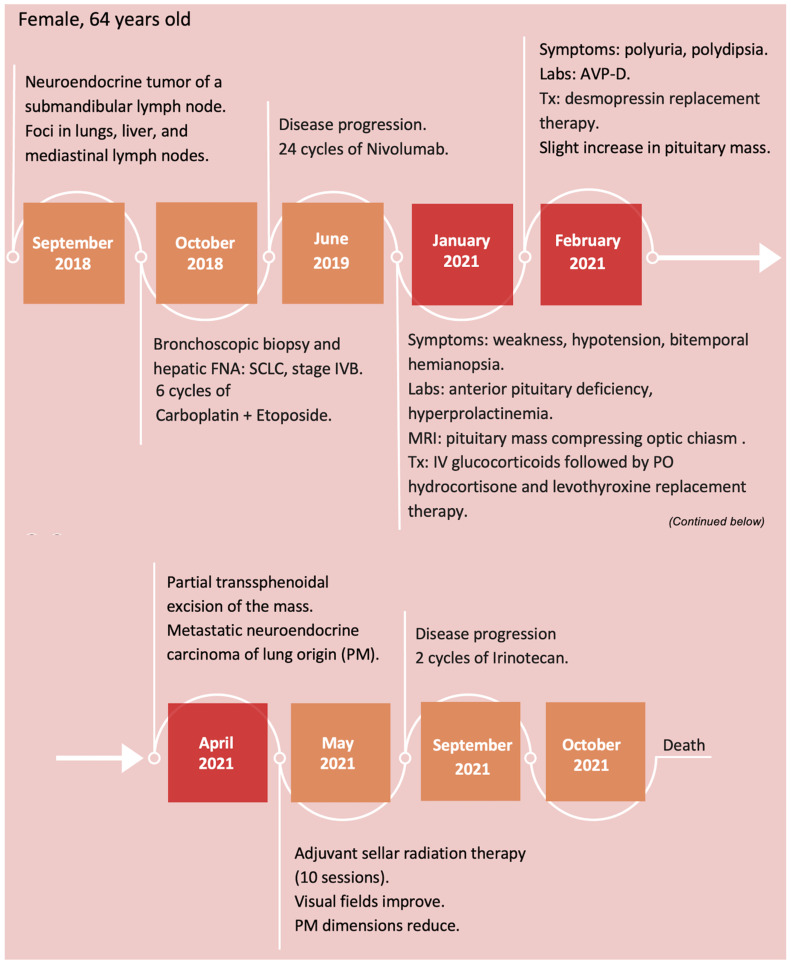
Case 1 as a timeline. The time of PM diagnosis is indicated in red. FNA; fine needle aspiration, SCLC; small cell lung carcinoma, AVP-D; arginine-vasopressin deficiency, Tx; treatment, IV; intravenously, PO; per os, PM; pituitary metastasis.

**Figure 3:**
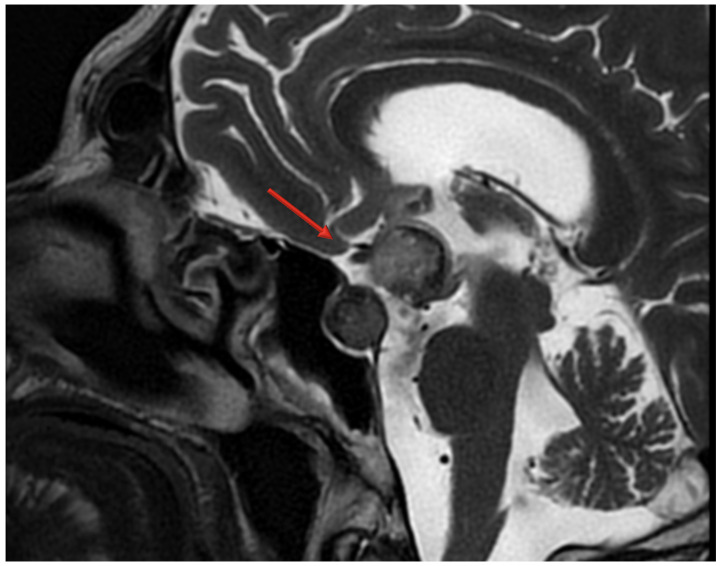
Sagittal T2-weighted brain MRI in Case 2. Dumbbell-shaped lesion (red arrow) centered in the pituitary sella, measuring ~21 × 14 mm, with suprasellar extension and compression of the optic chiasm.

**Figure 4:**
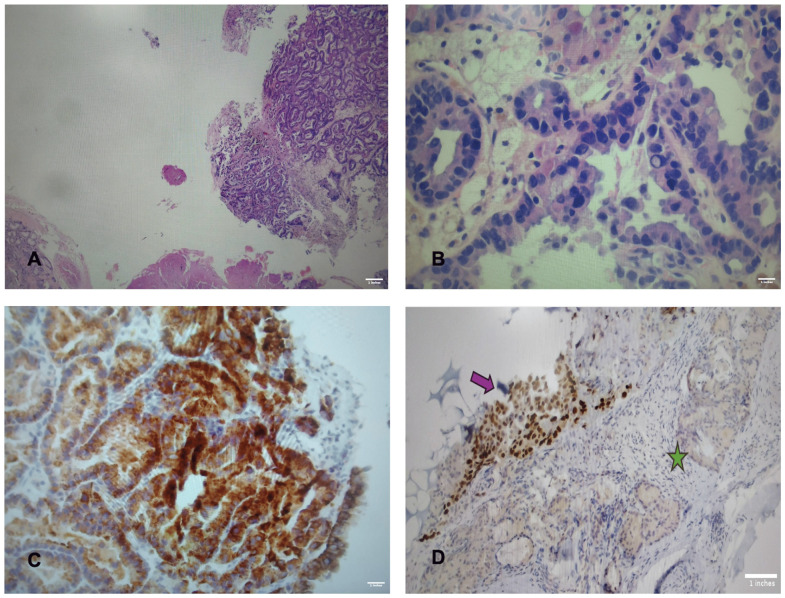
Histopathological and immunohistochemical features of the pituitary tumor in Case 2 (scale bar: 1 inch). (**A**) H&E stain (×20) showing fibrous meningeal tissue intermixed with respiratory epithelium. (**B**) H&E stain (×400) highlighting malignant glandular structures within the lesion. (**C**) Napsin A immunostain (×200) demonstrating focal, intense positivity in glandular cells. (**D**) TTF-1 immunostain (×40) showing strong nuclear positivity (purple arrow); background inflammatory infiltrate is marked by a (green asterisk).

**Figure 5:**
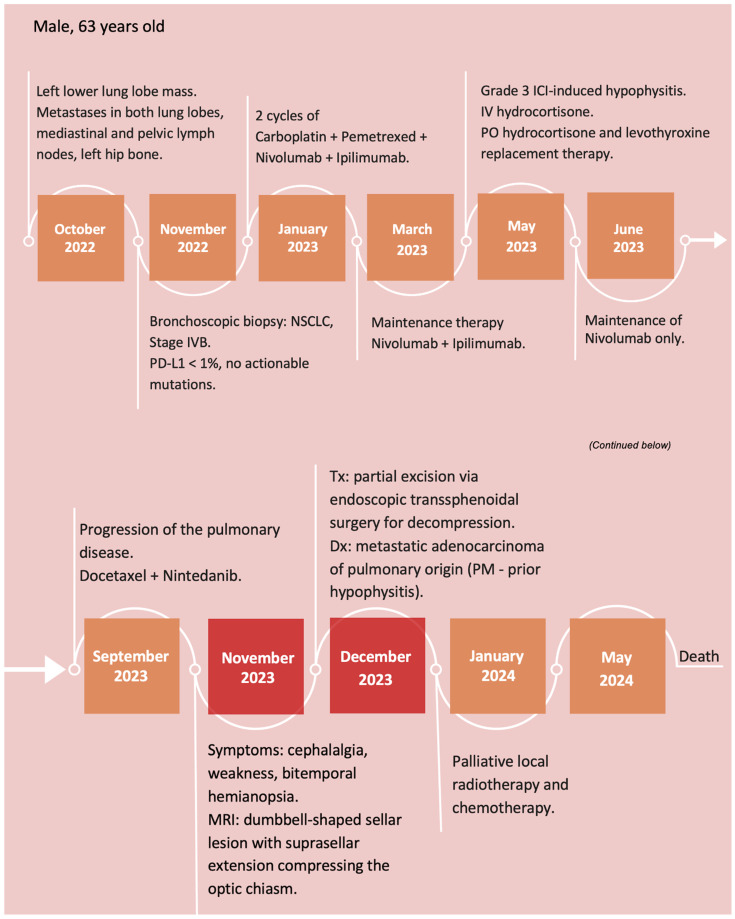
Case 2 as a timeline. The time of PM diagnosis is indicated in red. NSCLC; non-small cell lung carcinoma, PD-L1; programmed death-ligand 1, ICI; immune-checkpoint inhibitors, IV; intravenously, PO; per os, Tx; treatment, Dx; diagnosis.

**Figure 6:**
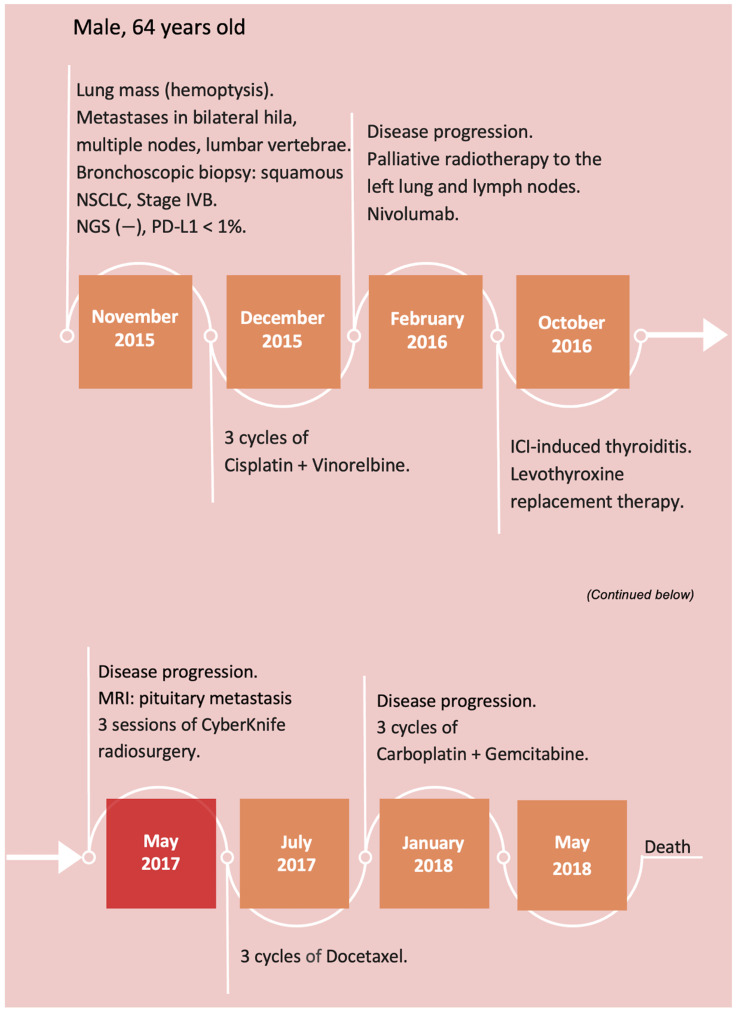
Case 3 as a timeline. The time of PM diagnosis is indicated in red. NSCLC, non-small cell lung carcinoma; NGS, next-generation sequencing; PD-L1, programmed death-ligand 1; ICI, immune-checkpoint inhibitor.

**Figure 7:**
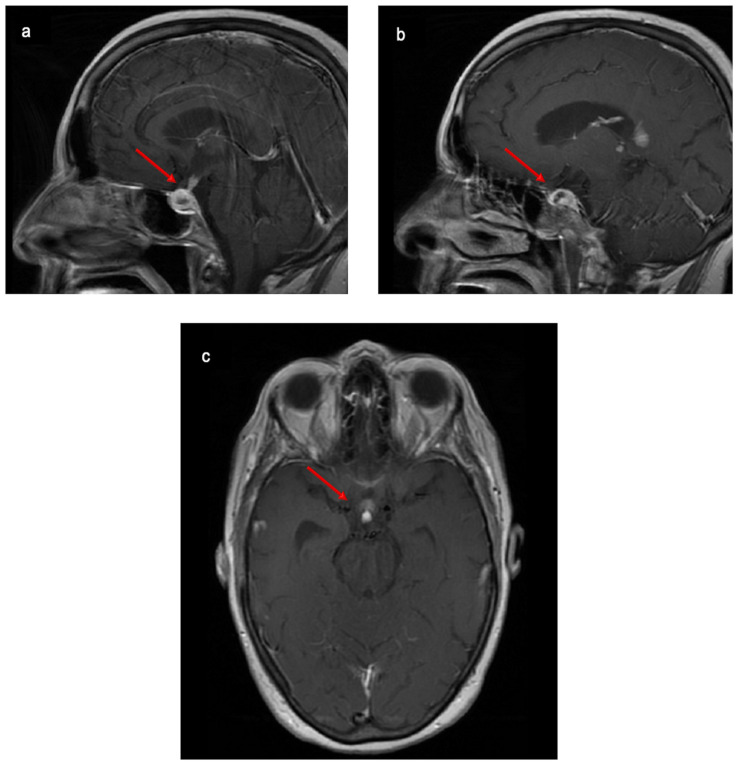
Sagittal (**a**,**b**) and axial (**c**) T1-weighted brain MRI in Case 4. Pituitary mass occupying the sellar region with suprasellar extension and displacement of the optic chiasm (red arrow).

**Figure 8:**
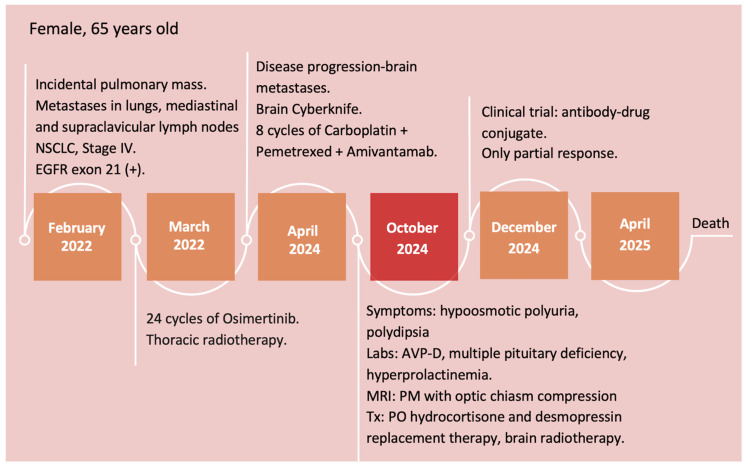
Case 4 as a timeline. The time of PM diagnosis is indicated in red. NSCLC; non-small cell lung carcinoma, EGFR; epidermal growth factor receptor, AVP-D; arginine-vasopressin deficiency, PM; pituitary metastasis, Tx; treatment, PO; per os.

**Table 1:** Patients’ demographic and disease characteristics.

Case	Gender	Initial Diagnosis	Age at LC Diagnosis	Stage at Diagnosis	PM Presentations	Pituitary Insufficiency	MRI Findings	OS (Months)	Survival After PM (Months)
1	Female	Neuroendocrine tumor (SCLC)	64	IV	Weakness, hypotension, visual field defects, polydipsia, polyuria	Anterior and posterior	Pituitary mass (maximum diameter, 1.5 cm), suprasellar extension, compression of the optic chiasm	43	6
2	Male	Adenocarcinoma (NSCLC)	63	IV	Headaches, dizziness, weakness, visual field defects	Anterior	Pituitary dumbbell-shaped tumor (21 × 14 mm), suprasellar extension, compression of the optic chiasm	18	6
3	Male	Squamous cell carcinoma (NSCLC)	64	IV	Asymptomatic	-	Pituitary mass (maximum diameter, 1.3 cm), extension to the left cavernous sinus	26	12
4	Female	Adenocarcinoma (NSCLC)	65	IV	Polydipsia, polyuria	Anterior and posterior	Pituitary mass (1.8 × 0.8 cm), compression of the optic chiasm	37	6

SCLC; small cell lung cancer, NSCLC; non-small cell lung cancer, MRI; magnetic resonance imaging, PM; pituitary metastasis, OS; overall survival.

**Table 2:** Laboratory exams of the patients at the time of pituitary metastasis diagnosis. Normal values are shown in brackets.

Laboratory Test	Case 1	Case 2	Case 3	Case 4
TSH (μIU/mL)	0.01 (0.35–4.45)	0.18 (0.35–4.45)	2.82 (0.35–4.45)	<0.01 (0.35–4.45)
FT4 (ng/dL)	1.35 (0.93–1.7)	0.64 (0.93–1.7)	1.14 (0.93–1.7)	1.08 (0.93–1.7)
LH (mIU/mL)	<0.1 (7.7–58.5)	<0.1 (1.7–8.6)	2.3 (1.7–8.6)	<0.1 (7.7–58.5)
FSH (mIU/mL)	1.49 (26.72–133.41)	0.8 (1.5–12.4)	-	0.76 (26.72–133.41)
TESTO (ng/mL)	-	1.2 (1.93–7.4)	2.3 (1.93–7.4)	-
ACTH (pg/mL)	3.5 (6–69)	5 (6–69)	15.8 (6–69)	2.7 (6–69)
Cortisol (μg/dL)	12.96 (6.2–19.4)	29 (6.2–19.4)	12 (6.2–19.4)	0.7 (6.2–19.4)
GH (ng/mL)	-	0.09 (<5)	-	1.4 (<5)
PRL (ng/mL)	55.28 (5.2–26.5)	13.1 (3.5–19.4)	-	36.13 (5.2–26.5)
IGF-1 (ng/mL)	136.9 (30–230)	52.8 (30–230)	-	123 (30–230)
24 h urine volume (mL)	8500	-	-	9700
Urine Osmolality (mOsm/kg)	120 (150–1150)	-	-	102 (150–1150)
Serum Osmolality (mOsm/kg)	310 (275–295)	-	-	312 (275–295)

TSH; thyroid-stimulating hormone, FT4; free thyroxine, LH; luteinizing hormone, FSH; follicle-stimulating hormone, TESTO; testosterone, ACTH; adrenocorticotropic hormone, GH; growth hormone, PRL; prolactin, IGF-1; insulin-like growth factor-1, (-); not available. Note: Reference ranges for hormonal assays vary across cases due to sex-specific differences and the utilization of various external laboratories for specialized tests not available at the primary institution (Sotiria Hospital).

## Data Availability

The original contributions presented in this study are included in the article. Further inquiries can be directed to the corresponding author.

## Abbreviations

The following abbreviations are used in this manuscript:

ACTH	Adrenocorticotropic Hormone
AVP-D	Arginine Vasopressin Deficiency
CK	Cytokeratin
CT	Computed Tomography
CTLA-4	Cytotoxic T-Lymphocyte-Associated Protein 4
ECOG	Eastern Cooperative Oncology Group
EGFR	Epidermal Growth Factor Receptor
FSH	Follicle-Stimulating Hormone
FT4	Free Thyroxine
GATA3	GATA Transcription Factor 3
GH	Growth Hormone
H&E	Hematoxylin and Eosin
ICI	Immune Checkpoint Inhibitor
IGF-1	Insulin-Like Growth Factor-1
LC	Lung Cancer
LH	Luteinizing Hormone
MRI	Magnetic Resonance Imaging
NSCLC	Non-Small Cell Lung Cancer
PD-L1	Programmed Death-Ligand
PET/CT	Positron Emission Tomography/Computed Tomography
Pit1	Pituitary-Specific Positive Transcription Factor 1
PM	Pituitary Metastasis
PRL	Prolactin
RECIST	Response Evaluation Criteria in Solid Tumors
SCLC	Small Cell Lung Cancer
SF-1	Steroidogenic Factor 1
T-PIT	T-Box Transcription Factor
TSH	Thyroid-Stimulating Hormone
TTF-1	Thyroid Transcription Factor-1
